# Comprehensive Peri-Operative Risk Assessment and Management of Geriatric Patients

**DOI:** 10.3390/diagnostics14192153

**Published:** 2024-09-27

**Authors:** Nikolaos Theodorakis, Maria Nikolaou, Christos Hitas, Dimitrios Anagnostou, Magdalini Kreouzi, Sofia Kalantzi, Aikaterini Spyridaki, Gesthimani Triantafylli, Panagiota Metheniti, Ioannis Papaconstantinou

**Affiliations:** 1 School of Medicine, National, and Kapodistrian University of Athens, 75 Mikras Asias, 11527 Athens, Greece; n.theodorakis@flemig-hospital.gr; 2 Department of Cardiology, Sismanogleio-Amalia Fleming General Hospital, 14 25is Martiou Str., 15127 Melissia, Greece; ch.chitas@flemig-hospital.gr (C.H.); jimdimitris100@gmail.com (D.A.); 3 Geriatric Outpatient Clinic, Sismanogleio-Amalia Fleming General Hospital, 14 25is Martiou Str., 15127 Melissia, Greece; magdalinikreouzi1998@yahoo.com (M.K.); sofia_kalanji@yahoo.gr (S.K.); kspyridaki@yahoo.gr (A.S.); manifilli@gmail.com (G.T.); 4 Department of Internal Medicine, Sismanogleio-Amalia Fleming General Hospital, 14 25is Martiou Str., 15127 Melissia, Greece; 5 Second Department of Surgery, Aretaieion General Hospital, National and Kapodistrian University of Athens, 76 Vasilissis Sofias Ave., 11528 Athens, Greece; panagiotameth@gmail.com (P.M.); johnpapacon@med.uoa.gr (I.P.)

**Keywords:** comprehensive geriatric assessment, peri-operative risk assessment, rehabilitation, prehabilitation, nutrition, exercise, elderly, frailty, sarcopenia

## Abstract

**Background:** As the population ages, the prevalence of surgical interventions in individuals aged 65+ continues to increase. This poses unique challenges due to the higher incidence of comorbidities, polypharmacy, and frailty in the elderly population, which result in high peri-operative risks. Traditional preoperative risk assessment tools often fail to accurately predict post-operative outcomes in the elderly, overlooking the complex interplay of factors that contribute to risk in the elderly. **Methods:** A literature review was conducted, focusing on the predictive value of CGA for postoperative prognosis and the implementation of perioperative interventions. **Results:** Evidence shows that CGA is a superior predictive tool compared to traditional models, as it more accurately identifies elderly patients at higher risk of complications such as postoperative delirium, infections, and prolonged hospital stays. CGA includes assessments of frailty, sarcopenia, nutritional status, cognitive function, mental health, and functional status, which are crucial in predicting post-operative outcomes. Studies demonstrate that CGA can also guide personalized perioperative care, including nutritional support, physical training, and mental health interventions, leading to improved surgical outcomes and reduced functional decline. **Conclusions:** The CGA provides a more holistic approach to perioperative risk assessment in elderly patients, addressing the limitations of traditional tools. CGA can help guide surgical decisions (e.g., curative or palliative) and select the profiles of patients that will benefit from perioperative interventions to improve their prognosis and prevent functional decline.

## 1. Introduction

The global demographic landscape is shifting, with an increasing proportion of people reaching older ages. By 2050, one in six people globally and one in four in Europe and Northern America will be 65+ years. Additionally, the global number of individuals aged 80+ years is projected to triple by 2050 [1]. Elderly patients are characterized by an increased prevalence of various conditions, including diseases that require surgical management, such as malignancies and ophthalmologic, general surgical, and orthopedic procedures. As a result, over 50% of surgical procedures are performed on patients aged 65+ years [2].

Geriatric patients are often characterized by unique characteristics, such as multimorbidity, polypharmacy, frailty, sarcopenia, and cognitive dysfunction, which increase their vulnerability to peri-operative complications [3]. Furthermore, the current tools for preoperative assessment often fail to accurately predict the peri-operative outcomes in elderly patients, especially because such tools do not take into account their unique characteristics that affect biological age. Coupled with the frequent need for surgical management in this continuously growing population, there is an increased demand to implement a specialized Comprehensive Geriatric Assessment (CGA) in the preoperative setting. This assessment considers several factors, including frailty, sarcopenia, nutritional status, cognitive function, mental health, and functional status and can be more predictive regarding the post-operative outcomes, including the occurrence of functional decline [4]. 

This review aims to provide a comprehensive overview of peri-operative risk assessment and management for geriatric patients. Such an approach aims to determine the cost-effectiveness and guide the selection of surgical procedure—curative or palliative—in elderly subgroups with specific characteristics, such as extreme age, severe frailty, and advanced dementia. In the current era, the decision to undergo surgery is largely patient-centered, with the ultimate choice lying with the patient. However, this decision is heavily influenced by the information and recommendations provided by their doctors. Therefore, a comprehensive peri-operative assessment that can accurately predict peri-operative outcomes is crucial to properly inform patients during the shared decision-making process. Furthermore, this assessment can reveal specific groups of patients who may be candidates for preoperative and post-operative rehabilitation to improve their short- and long-term outcomes. 

## 2. Surgical Procedures in the Elderly

Elderly patients are frequently subjected to a variety of surgical procedures. According to a recently published observational study among 2110 middle-aged and elderly patients with a mean age of 63.7 years, the top five most performed surgical operations were major joint surgery (18.1%), ophthalmological surgery (12.4%), abdominal surgery (10.2%), cosmetic surgery (8.8%), and foot or leg surgery (6.8%) [5]. 

Orthopedic surgical procedures, especially hip and knee replacements, are very common among the elderly due to the increasing prevalence of osteoarthritis with age. In 2022, the average age of a total knee replacement patient was 67.2 years old, while the average total hip replacement patient was 65.7 years old [6]. Notably, 51.4% of the totality orthopedic procedures are performed on the elderly [2]. Ophthalmological surgery, especially cataract surgery, is the most common surgical intervention in the elderly. A study has shown that 88% of ophthalmological surgical procedures are performed on the elderly [2]. 

General surgical operations represent a major group of surgeries that are commonly performed on the elderly. These procedures include colectomy, pancreatectomy, hepatectomy, cholecystectomy, breast surgery, and hernia repair. A study has shown that geriatric patients represent 5.6% of candidates in general surgical procedures [2]. Regarding colorectal cancer, a very common malignancy in the elderly, a study has shown that among 1 million patients 63.8% of the operations were performed on patients 65 years and older and 22.6% on patients 80 years and older [7]. Additionally, urological and gynecological procedures are commonly performed on the elderly, as there is increasing prevalence of benign prostatic hyperplasia, pelvic prolapse, and urological/gynecological malignancies with age. A study has shown that 64.8% of urological procedures are performed on geriatric patients [2]. 

Cardiothoracic operations, including coronary artery bypass grafting and valve repair or replacement, are common due to the high prevalence of cardiovascular diseases in aged populations. Studies has shown that the elderly account for 43–70% of candidates in such operations [2,8]. Finally, neurosurgical procedures, including tumor resection and shunt placements, are additional operations that are occasionally performed on the elderly, who represent 45.2% of the candidates for such operations [2]. 

## 3. Post-Operative Complications in the Elderly

Despite advances in surgical techniques and peri-operative care, post-operative complications are still common in the elderly, possibly due to their increased burden of comorbidities and other factors such as frailty. Examples of such complications include infections, thromboembolic events, post-operative delirium (POD), and functional decline. These lead to prolonged hospital length of stay (LOS), increased peri-operative mortality, patient distress, and increased health costs. 

Results from a large multi-center study (*n* = 568,263, 26,648 of which are 80+ years) showed that the incidence of at least one post-operative complication is 20% in patients 80+ years, compared to 12.1% in patients 0–79 years (*p* < 0.001). From the same study, the incidence of post-operative urinary tract infections and pneumonia was 5.6% and 5.6% in patients 80+ years, compared to 2.2% and 2.3% in patients 0–79 years, respectively (*p* < 0.001 for all comparisons). The post-operative 30-day mortality in patients with at least one complication is 26.1% in patients 80+ years and 15,1% in patients 0–79 years (*p* < 0.001). At the same time, among patients without post-operative complications, the mortality was higher in those 80+ years (3.7%) compared to those 0–79 years (1.1%) (*p* < 0.001) [9]. 

Results from a recent meta-analysis showed that POD is another significant complication in the elderly, with an incidence of 7–56% among patients 65+ years. POD is commonly associated with anesthesia, while significant risk factors include major surgeries, frailty, dementia, prolonged hospital stay, and the presence of other complications [10]. 

Other common post-operative complications that are more frequent among the elderly include wound complications and surgical-site infections, bleeding complications, venous thromboembolism, prolonged ileus, acute kidney injury, arrhythmias, acute coronary syndromes, pulmonary edema, and cerebrovascular events. Most post-operative complications occur within 30 days of the operation and pose the most important determinants of mortality in surgical patients [9,11]. 

A retrospective cohort by Finlayson et al. showed that post-operative mortality for major oncological surgeries was approximately double for octogenarians compared to patients aged 65–69 years. Another finding of the study was that the presence of multi-morbidity was associated with lower survival rates among octogenarians [12]. Furthermore, 24–44% of octogenarians who were discharged after a successful operation demonstrated significant functional decline and required institutionalization. 

Long-term outcomes for elderly surgical patients are also concerning. A prospective cohort by Khuri et al. demonstrated that old age and functional status are two of the most important risk factors that predict long-term prognosis after surgery [13]. 

These studies demonstrate the impact of post-operative complications among the elderly, highlighting the need to optimize peri-operative assessment and management to improve their outcomes. 

## 4. Tools for Traditional Preoperative Risk Assessment

Several traditional risk stratification tools and models are available to assess peri-operative risk. Three of the most notable and widely used scores include the American Society of Anesthesiologists (ASA) classification system, the Revised Cardiac Risk Index (RCRI), and the American College of Surgeons National Surgical Quality Improvement Program (ACS NSQIP) Surgical Risk Calculator [14,15,16,17,18,19]. However, these tools overlook the unique characteristics and complexities of elderly patients and provide inaccurate estimations. 

The ASA classification is widely used to assess the physical status of patients before surgery. It categorizes patients into six classes based on their overall health and presence of comorbid conditions. While it provides a general overview, it is a subjective score with high inter-observer variability, and it may not capture all aspects of geriatric risk. Studies of the general population have shown that ASA classification is associated with multiple outcomes, including operative duration, post-operative complications, hospital LOS, intensive care unit admissions, and survival [14]. However, its accuracy in predicting peri-operative outcomes in elderly patients has been questioned. In one study of patients aged 70+ years who underwent colorectal surgery, ASA classification was not associated with post-operative complications [15]. Another study demonstrated that ASA classification was associated with major post-operative complications but not with hospital LOS or 30-day mortality in elderly patients undergoing various oncologic surgical procedures [16]. Additionally, a study of 108 patients aged 65–84 demonstrated that frailty and multiple CGA domains but not ASA were associated with post-operative morbidity [17]. Furthermore, the intermediate and possibly long-term prognosis of patients is poorly assessed with ASA compared to the CGA. Specifically, a study of 980 patients aged 75+ years who underwent oncological surgery demonstrated that 6-month mortality was only associated with the number of deficits in the CGA but not with ASA physical status [18].

The RCRI is specifically designed to evaluate post-operative cardiac risk. It considers factors, including high-risk surgery, history of ischemic heart disease, history of congestive heart failure, history of cerebrovascular disease, preoperative insulin therapy, and renal dysfunction [19]. However, it does not account for frailty or other geriatric-specific factors, limiting its predictive accuracy in elderly populations. A study demonstrated that the RCRI has inferior accuracy in the elderly compared to a more comprehensive assessment that includes functional status [20]. 

The ACS NSQIP Surgical Risk Calculator incorporates patient-specific factors to estimate the risk of post-operative complications. However, its utility in predicting outcomes for geriatric patients is limited by its focus on comorbidities rather than biological age. A study by van der Hulst et al. showed that, except for post-operative pneumonia, this score had a poor performance in predicting all other post-operative outcomes among elderly patients who underwent elective colorectal surgery [21]. 

As a result, traditional risk stratification tools often do not account for the biological age of patients, focusing instead on chronological age and specific comorbidities. This is evident by the aforementioned studies, which showed that these tools can be poor predictors of peri-operative outcomes in elderly patients’ aging. This highlights the importance of implementing a CGA, including parameters such as frailty and sarcopenia, which are often indicative of the patients’ functional status and biological age. As we will discuss in the next section, this strategy is more accurate in predicting the post-operative outcomes, and, at the same time, it is useful to plan for individualized peri-operative interventions and rehabilitation to improve both the prognosis and functional capacity of the elderly surgical patients. 

## 5. Preoperative Comprehensive Geriatric Assessment

### 5.1. General

The CGA is a multidimensional, interdisciplinary diagnostic process to determine the medical, psychological, and functional capabilities of elderly individuals. It is considered the gold standard for assessing geriatric patients and tailoring interventions to improve outcomes. The CGA typically includes assessments of various parameters, including medical comorbidities, presence of polypharmacy, presence of frailty, functional status, cognitive function, nutritional status, risk of falls, and psychosocial factors [4]. However, there is lack of studies determining the gold-standard tool for the CGA in elderly patients, especially in the context of a preoperative assessment. The above, together with the fact that a typical CGA is complex, time consuming, and requires specialized personnel, limit its use in everyday practice in the context of a preoperative assessment. In this section, we will provide an overview of the tools within a typical CGA, while we will focus on providing current evidence regarding its predictive value regarding post-operative outcomes and the planning of peri-operative interventions. In this way, we can encourage further research, aiming to determine the gold-standard tool for performing a preoperative CGA in a simple and timely manner so it can be integrated into everyday surgical practice. The components of a preoperative CGA and their prognostic roles are presented in Table 1.

### 5.2. Frailty

Frailty is a key component of the CGA and is a strong predictor of adverse surgical outcomes. Frailty is a common syndrome among the elderly, characterized by progressive functional decline, decreased physiological reserve, and reduction in the resistance to endogenous and exogenous stressors, leading to an increase in an individual’s vulnerability [22]. Frailty could reconcile the difference between a patients’ chronological and biological age. It is generally proposed that frailty is objectively assessed using sets of predefined criteria rather than the physician’s eyeball estimation. Even though there is no gold-standard method to assess frailty, there are several tools that can be used in clinical practice. Some of these tools are extensive, being time consuming and requiring expertise, while others are easily applied but less comprehensive. One of the most widely recognized criteria for diagnosing frailty is the Fried phenotype, derived from the Cardiovascular Health Study and the Women’s Health and Aging Study. This approach identifies pre-frailty by the presence of one or two criteria and frailty by the presence of three or more criteria out of five: weight loss, exhaustion, low grip strength, low gait speed, and low activity level [20]. Frailty can also be assessed as an accumulation of deficits (e.g., organic comorbidities, cognitive decline, malnutrition, and psychosocial issues) contributing to the patient’s overall vulnerability. The more deficits an individual accumulates, the higher their level of frailty and the greater their risk for adverse outcomes, such as falls, hospitalization, and mortality. In this context, frailty can be assessed using specific deficit accumulation indexes, such as the Frailty Index by Rockwood [23]. 

The available evidence from meta-analyses and prospective cohorts is suggestive that preoperative frailty is a strong risk factor for adverse clinical outcomes following elective and emergent general surgical procedures in elders, including the increased incidence of post-operative complications, prolonged hospital LOS, and increased 30-day, 90-day, and 1-year mortality [24]. A large meta-analysis (*n* = 1,153,684 patients aged 58–85 years) showed that frail patients, compared to non-frail, had odd ratios (OR) of 2.56, 5.77, and 2.71 for post-operative complications and short- and long-term mortality, respectively [25]. Because aging could be a confounder, a meta-regression analysis in the above study revealed that age per se did not increase the risk of major post-operative morbidity and short-term mortality. Another meta-analysis (*n* = 2281 aged 61–77 years) showed that 30-day mortality in frail and pre-frail patients was 8%, compared to 1% in non-frail patients. In the same study, the incidence of post-operative complications was 24%, 9%, and 5% in frail, pre-frail, and non-frail individuals, respectively, while the hospital LOS was 9.6 days in frail and 6.4 days in non-frail patients [26]. A recent meta-analysis concluded that frail patients, compared to non-frail, had an OR of 2.32 (data from case-control studies) or a relative risk (RR) of 3.64 (data from prospective cohorts) for POD [27]. 

### 5.3. Sarcopenia

Beyond frailty, another distinctive characteristic of old age is sarcopenia, which is commonly assessed in the context of a CGA. Sarcopenia is a progressive and generalized skeletal muscle disorder that involves the accelerated loss of muscle mass and strength. It is primarily associated with aging but can also be exacerbated by physical inactivity, chronic diseases, and malnutrition [28]. Sarcopenia significantly contributes to physical disability, poor quality of life, and increased mortality in the elderly. 

The pathophysiology of sarcopenia is multifactorial and involves several complex processes. One of the hallmark features of sarcopenia is the denervation and selective loss of type 2 (fast-twitch) muscle fibers, which are primarily responsible for generating power and speed. As these motor neurons degenerate, the muscle fibers they innervate either undergo atrophy or are reinnervated by surviving neurons, which are often associated with type 1 fibers (type 2 to type 1 shift) [28]. Additionally, chronic low-grade inflammation, often referred to as “inflammaging”, plays a significant role in the development of sarcopenia. Elevated levels of pro-inflammatory hormones can promote muscle catabolism and impair muscle protein synthesis. Furthermore, hormonal changes, including a decline in anabolic hormones such as testosterone and growth hormone, as well as insulin resistance, are key mediators of sarcopenia in the elderly. Another mechanism that contributes to sarcopenia is age-related mitochondrial dysfunction, which leads to ATP shortage, oxidative stress, and the accumulation of misfolded proteins within muscle cells, causing dysfunction and apoptosis. As the muscle mass decreases, there is often a concomitant increase in intramuscular fat and fibrous connective tissue. This process, known as myosteatosis, contributes to the decline in muscle quality and function and promotes inflammation and insulin resistance, creating a vicious cycle [28]. 

Initial methods for assessing sarcopenia include measurement of grip strength as well as the chair stand test. Methods to confirm the presence of sarcopenia include the use of dual-energy X-ray absorptiometry, bioelectrical impedance analysis, or measuring muscle mass using imaging techniques. The severity of sarcopenia can be assessed with various methods, including gait speed, Short Physical Performance Battery (SPPB), and the Timed Up and Go (TUG) test [29]. Assessing sarcopenia helps identify patients who may benefit from peri-operative nutritional support and physical training interventions. 

Multiple studies have identified sarcopenia as a predictive marker for post-operative outcomes. A meta-analysis by Wang et al. demonstrated that the presence of sarcopenia in elderly patients is associated with a 4-fold increased risk of post-operative complications, a more than 2-fold increased risk in 30-day mortality, and a prolonged hospital LOS by an average of 4.5 days [30]. Another study by Lieffers et al. demonstrated in post-operative cancer patients that the presence of sarcopenia was associated with increased post-operative infections (by 11.2%), prolonged hospital LOS (by 1.7 days), and increased needs for in-patient rehabilitation (by 8.7%) [31]. Additionally, Reisinger et al. showed that sarcopenia in patients undergoing surgery for colorectal cancer was associated with an increased post-operative inflammatory response [32].

### 5.4. Nutritional Status

Nutritional status is a critical component of health in the elderly, influencing physical and cognitive function, immune response, and overall quality of life. Malnutrition is prevalent among older adults and can adversely affect peri-operative outcomes. Causes of malnutrition in the elderly include dental issues, dysphagia, gastrointestinal changes, chronic diseases, medications, psychological factors, cognitive impairment, and socioeconomic factors. Nutritional assessment is a crucial component of the CGA [33]. The Mini Nutritional Assessment (MNA) is one of the most widely used screening tools for assessing nutritional status in the elderly. The MNA evaluates factors such as weight loss, appetite, mobility, psychological stress, neuropsychological problems, and body mass index (BMI). It also considers dietary intake, including the consumption of protein-rich foods and fluids. The Malnutrition Universal Screening Tool (MUST) is another commonly used screening tool that assesses nutritional risk based on BMI, unintentional weight loss, and the presence of acute disease that may lead to poor nutritional intake [33]. Assessing malnutrition helps identify patients who may benefit from peri-operative nutritional support and physical training interventions. 

Malnutrition is an important risk factor for adverse peri-operative outcomes. A study by Millrose et al. demonstrated that malnutrition in geriatric patients increases the risk of post-operative mortality (up to 2-fold) and is associated with up to a 1.5-fold risk of worsened mobility and independence in patients with hip fractures [34]. Additionally, Buzby et al. demonstrated that malnourished patients undergoing gastrointestinal surgical operations had up to a 46% incidence of complications [35]. Similarly, Akula et al. showed that malnutrition in surgical patients undergoing gastrointestinal operations is associated with a 2.41-fold increased risk for post-operative complications, especially infections, and an impressive 11.5-fold higher mortality [36]. 

### 5.5. Cognitive Function and Mental Health

Cognitive dysfunction is common among the elderly. It encompasses a range of disorders that can vary in severity from mild cognitive impairment to dementia. The most common causes include Alzheimer’s disease, vascular dementia, frontotemporal dementia, Lewy body dementia, Parkinson’s disease, and normal pressure hydrocephalus [37]. Cognitive dysfunction can significantly affect the individuals’ independence, mood, social life, sleep patterns, and overall quality of life. Advanced dementia is also associated with dysphagia, malnutrition, increased risk of aspiration, immobilization and pressure ulcers, increased risk for hospitalization, and death [37]. Cognitive dysfunction is particularly concerning in the peri-operative setting, where it can increase the risk of POD, prolong hospital LOS, and lead to increased mortality. 

Tools used to assess the presence of cognitive dysfunction in the context of a CGA include the Mini Mental State Examination (MMSE), the Montreal Cognitive Assessment (MoCA), and the clock drawing test [38]. The MMSE is one of the most widely used tools for screening cognitive function. It assesses various cognitive domains, including orientation, registration, attention, calculation, recall, language, and the ability to follow simple commands. Scores range from 0 to 30, with 24 being a commonly used cut-off. The MoCA is another widely used screening tool, particularly for detecting mild cognitive impairment. It assesses a broader range of cognitive domains than the MMSE, including executive function, visuospatial abilities, and attention, making it more sensitive for detecting early cognitive changes. Similarly, the clock drawing test is another simple tool used to detect mild cognitive impairment. The patient is asked to draw a clock showing a specific time, which is then scored based on the accuracy of the drawing [38]. 

Mental health disorders are quite common among the elderly, with the two leading diagnoses being major depressive disorder and generalized anxiety disorders with significant overlap. These conditions can significantly affect the quality of life, increase the risk of substance abuse, and decrease the adherence to medications [39]. An assessment of mental health remains a cardinal aspect of a CGA, which is frequently performed via the use of questionnaires, typically supplemented with a formal assessment by a mental health professional. The Geriatric Depression Scale (GDS) is a screening tool specifically designed to identify symptoms of depression in older adults. It was created to be simple and easy to use, and it features a series of 30 questions that users answer in a “Yes/No” format [40]. 

Multiple studies have identified the presence of cognitive dysfunction as a predictive marker for post-operative outcomes. A study by Saczynski et al. found that preoperative cognitive impairment was an independent predictor of POD in elderly patients undergoing cardiac surgery. Notably, the development of POD was associated with a significant decline in cognitive function during the first year after surgery [41]. A retrospective study by Blair et al. demonstrated that patients with dementia had a 1.5-fold increase in the risk of 90-day mortality and readmission. On the other hand, the same study showed that patients with mild cognitive impairment were less likely to undergo elective surgery, even though they had similar outcomes as patients with normal cognition [42]. Additionally, a study by Tsuda et al. showed that the presence of dementia was associated with a 1.45-fold increase in the risk of post-operative complications after hip fracture surgery, especially surgical site infections, urinary tract infections, and respiratory complications [43].

### 5.6. Functional Status

Functional status is a critical predictor of surgical outcomes in elderly patients. It reflects an individual’s ability to perform basic and complex activities necessary for daily living. Assessing functional status helps clinicians understand a patient’s baseline physical capacity and identify those at risk for post-operative complications, prolonged recovery, or loss of independence. Given its importance, functional status assessment is a fundamental component of the CGA [44]. The Barthel Index is a widely used tool for assessing the ability to perform basic activities of daily living (ADLs). It evaluates ten domains, including feeding, bathing, grooming, dressing, bowel and bladder control, toilet use, transfers, mobility, and stair climbing. The Katz Index is another tool used to assess basic ADLs. It focuses on six key functions: bathing, dressing, toileting, transferring, continence, and feeding. The Lawton IADL Scale assesses more complex activities that are essential for living independently in the community. These activities include using the telephone, shopping, food preparation, housekeeping, laundry, transportation, medication management, and handling finances. The Lawton scale is particularly useful for identifying subtle declines in function that may not be apparent in ADL assessments [44]. 

Patients with poor functional status have poor post-operative outcomes. These patients are usually characterized by frailty and cognitive impairment, which are also predictors of worse outcomes, as already described. A study by Uchinaka et al. showed that patients with a Barthel Index <100 had a 3-fold increased risk of post-operative complications as well as an average of 5 day longer duration of hospital stay [45]. A meta-analysis by Chen et al. demonstrated that there is a high incidence of IADL dependence in older surgical patients undergoing non-cardiac and cardiac surgery, which was associated with a 2-fold increased risk of POD [46]. 

Initial methods for assessing sarcopenia include measurement of grip strength as well as chair stand test. Methods to confirm the presence of sarcopenia include the use of dual-energy X-ray absorptiometry, a bioelectrical impedance analysis, or by measuring muscle mass using imaging techniques. The severity of sarcopenia can be assessed with various methods, including gait speed, SPPB, and the TUG test [29]. Assessing sarcopenia helps identify patients who may benefit from peri-operative nutritional support and physical training interventions.

### 5.7. Implementation of Preoperative CGA in Everyday Practice

Several studies have demonstrated that implementation of a CGA can accurately predict post-operative outcomes and reveal profiles for specific patients who are candidates for prehabilitation and post-operative interventions aiming to improve the prognosis. Specifically, a study of 217 patients aged 75+ years with esophageal cancer demonstrated that patients with deficits in 2 or more domains in the preoperative CGA had statistically significantly higher rates of post-operative pneumonia and anastomotic leakage and a 1.38-fold higher risk of death [47]. Another study of 74 patients aged 80+ years with colorectal cancer showed that those with multiple deficits had a 2.36-fold risk of medical complications, a 3.3-fold higher risk of delirium, longer hospital LOS, a 2.38-fold higher risk of readmission, 3.3-fold higher 6-month mortality, and 3.28-fold higher 12-month mortality [48]. According to a systematic review, the majority of CGA domains are predictive of adverse post-operative outcomes, while frailty seems to be the most important predictor [49].

Despite the benefits, implementation of a complete CGA in everyday surgical practice can be complex, time-consuming, and require specialized personnel. A survey of the surgical task force at the International Society of Geriatric Oncology has demonstrated that among 251 surgeons who responded, only 6.4% performed a CGA in daily practice [50]. In this context, the International Society of Geriatric Oncology has recommended a two-step approach. This approach includes using a short screening tool that can be simple and can potentially be performed in a timely manner by non-specialized personnel after a short demonstration, followed by an extensive CGA in selected patients. An example of such screening method is the G8 Geriatric Screening tool [51]. The G8 tool includes the patients’ age and seven questions that can easily be asked by non-specialized physicians and nurses that assess nutrition, weight, mobility, neuropsychological conditions, polypharmacy, and perceived health status. A study utilizing the G8 tool for preoperative evaluation prior to major oncological abdominal surgery demonstrated that it could predict post-operative complications and 1-year mortality while it prompts further evaluation with a CGA [52].

### 5.8. CGA and Surgical Decision

None of the scores used for preoperative risk assessment, including traditional tools and CGAs, make explicit determinations about futility in the sense of definitively advising against surgery. Instead, they quantify risks and potential post-operative outcomes but leave the ultimate decision to the clinical judgment of the healthcare team, patient preferences, and ethical considerations. Futility in surgery is more nuanced and involves discussions around quality of life, life expectancy, functional outcomes, and the choice of the patient and relatives. The concept of futility generally comes into play when the likelihood of a positive outcome is extremely low or when the risks outweigh the potential benefits, particularly in terms of survival or functional recovery [53]. In this context, as already stated in the previous responses and analyzed within the manuscript, a CGA can be a more reliable tool for preoperative risk estimation compared to standard tools such as ASA, guiding the surgeon’s decision to operate and whether to choose a more palliative rather than curative approach. The major advantage the CGA offers is that it can detect abnormalities in specific domains (e.g., malnutrition and sarcopenia) that can potentially be corrected and improve post-operative outcomes, making surgery more successful, as analyzed in the next section of the manuscript.

## 6. Peri-Operative Management and Rehabilitation

### 6.1. Overview

Effective peri-operative management and rehabilitation is crucial to enhance recovery, minimize complications and functional decline, and improve overall survival rates of geriatric patients. In this final section, we will focus on nutritional interventions, physical training, physiotherapy, and correction of specific deficiencies, each supported by evidence demonstrating their efficacy in improving post-operative outcomes. Most studies that investigated the effects of peri-operative nutritional and physical interventions are not exclusive for the geriatric patients, which could be perceived as a limitation. However, the majority of participants in such studies are either elderly or frail, since this is the population in need of such interventions.

### 6.2. Nutritional Interventions 

Proper nutritional support is critical for geriatric patients undergoing surgery, given their susceptibility to malnutrition and its impact on surgical outcomes. Intervention studies have shown that nutritional supplementation, including high-protein diets and micronutrient-rich foods, can significantly reduce post-operative complications. 

A meta-analysis of 14 randomized controlled trials involving 611 patients—mostly aged 65+ years—who underwent various orthopedic surgeries showed that protein supplementation was associated with a significant reduction in muscle atrophy, particularly as measured by muscle cross-sectional area, with improvements noted in 11 of the 14 studies. For example, in the study by Dreyer et al. (patients with a mean age of 64.4 years), quadriceps muscle volume decreased by only 6.2% in the protein supplementation group compared to 18.4% in the control group. Additionally, functional improvements were observed, including quicker achievement of rehabilitation benchmarks, such as walking without aids or achieving full range of motion [54]. 

In a study by Fukuda et al. involving 800 patients with gastric cancer who underwent gastrectomy, among those with malnutrition (mean age of 73 years), patients who received nutritional support for at least 10 days experienced a lower rate of infections compared to those who had inadequate or no nutritional support [55]. According to Hsu et el., early initiation of oral nutrition following gastrectomy reduces the hospital LOS compared to delayed onset without an increase in complications when compared to patients who receive parenteral nutrition [56]. Additionally, in a cohort study by Gustafsson et al. (911 patients with a mean age of 69 years), nutritional intervention on the first post-operative day after colorectal cancer surgery was identified as an independent factor predicting 5-year survival rates [57]. 

A review by Delgado et al. highlights the significant impact of nutritional interventions following cardiac surgery [58]. Early enteral nutrition within 48 h post-surgery is associated with a significant reduction in mortality and complications. Patients receiving early enteral nutrition show a 20–30% reduction in infectious complications and a decrease in the duration of mechanical ventilation by 2–3 days compared to those without early nutritional support. When enteral nutrition was not feasible, early parenteral nutrition (started around day 3 post-surgery) can lead to a 15% reduction in ICU stays and a 25% improvement in muscle strength recovery. Overall, nutritional interventions can reduce the incidence of post-operative complications by 20–30%, emphasizing the critical role of nutritional support in improving patient outcomes [58]. 

A systematic review by Bao et al. found that early nutritional interventions significantly enhance post-operative outcomes in patients with complex fractures. The studies within the analysis primarily included elderly patients. Specifically, early nutritional support reduced REEDA (redness, edema, ecchymosis, discharge, and approximation) scores by 14.06 points one week post-surgery, indicating improved wound healing. Additionally, it lowered the Manchester Scar Scale scores by 25.03 points three months post-surgery, showing decreased scar formation [59]. 

### 6.3. Exercise and Physiotherapy

Aerobic and resistance training in the peri-operative period can improve recovery rates and reduce the duration of hospital stays. A recent systematic review by Piraux et al. investigated the peri-operative effects of preoperative combined aerobic and resistance training (prehabilitation) on cancer patients undergoing tumor resection surgery [49]. The analysis included 10 randomized controlled trials (360 patients with a mean age of 69 years). Physical capacity was improved in three out of five studies, and muscle strength reported improvements in two out of three studies in which these parameters were investigated. Quality of life improvements were noted in two out of the four studies in which it was investigated. Hospital LOS was reduced in one out of six studies. Post-operative complications, specifically respiratory complications, were lower in two out of six studies. None of the studies reported adverse effects from participating in exercise training, supporting the safety of such interventions in cancer patients [60]. 

A systematic review by Paul et al. investigated the impact of post-operative aerobic exercise training with or without resistance training on patients undergoing surgery for intra-abdominal cancers. The analysis included 11 studies, comprising 6 randomized controlled trials involving a total of 734 patients—the majority being 65+ years. Meta-analysis of four outpatient studies highlighted a significant improvement in the 6-min walk test (6 MWT), with a mean difference of 74.92 m. Quality of life assessments were varied across studies, with some showing significant improvements [61]. 

A systematic review by Stephensen et al. explored the effectiveness of preoperative and post-operative resistance exercise interventions on recovery of physical function in patients undergoing abdominal surgery for cancer. This review included 24 studies initially, with only 2 meeting the criteria for detailed analysis (73 patients). The preoperative intervention, tested on 42 patients (22 of whom were 65+), focused on resistance exercises for the lower limb and inspiratory muscle training, while the post-operative intervention, involving 31 patients, included a supervised program of resistance exercises targeting various muscle groups and stretching exercises [62]. Unfortunately, the study did not reveal any significant differences between the exercise and control groups in both arms. This could be attributed to the small sample size, highlighting the need for larger future studies. Furthermore, a careful selection of the appropriate candidates for resistance training, including patients with sarcopenia or malnutrition, might yield positive results in future studies. 

A meta-analysis by Boden et al. examined the effectiveness of preoperative physiotherapy in preventing post-operative pulmonary complications following major abdominal surgery. Analyzing data from 800 participants (mean age of 53 years) across two randomized controlled trials, the study found that preoperative physiotherapy reduced the risk of developing post-operative pulmonary complications by 47%. The benefits were particularly significant for very elderly, smokers, obese, and multimorbid patients. The analysis also showed a reduction in hospital LOS by 3.2 days on average for patients with multiple comorbidities but did not demonstrate a mortality benefit [63]. 

### 6.4. Management of Deficiencies 

A systematic review by Van Remoortel et al. assessed the efficacy of preoperative iron supplementation, with or without erythropoiesis-stimulating agents, on the need for red blood cell transfusion in patients with preoperative anemia undergoing elective surgery. The analysis included 29 randomized controlled trials and 2 non-RCTs with a large proportion of patients being 65+. The study found that intravenous or oral iron monotherapy may not significantly reduce the number of red blood cell units transfused. However, combining iron with erythropoiesis-stimulating agents reduced both the number of patients transfused and the number of RBC units used [64]. Another systematic review by Elhenawy et al. analyzed the effectiveness of preoperative intravenous iron therapy in reducing the need for blood transfusions in patients undergoing major surgery. The analysis incorporated 10 randomized controlled trials with a total of 1039 participants, the majority of whom were 65+ years. It demonstrated that preoperative intravenous iron significantly reduced blood transfusion requirements by 16% [65]. However, the effect of preoperative iron supplementation on post-operative complications and mortality remains inconclusive. 

A systematic review by Das and Bej evaluated the effect of vitamin D supplementation on post-operative outcomes in cardiac surgery patients. The review included 8 randomized controlled trials with a mean age of close to 65 years. The findings indicated that peri-operative vitamin D supplementation generally improved post-operative outcomes by normalizing vitamin D levels without adverse reactions, with 75% of the studies showing beneficial effects. About 37.5% of the studies reported a significant decrease in post-operative atrial fibrillation rates with vitamin D supplementation. Furthermore, some studies indicated a significant reduction in the need for inotropes during the post-operative period, while one study noted a marked reduction in the duration of ICU and hospital stays with vitamin D supplementation [66]. A meta-analysis conducted by Kuo-Chuan Hung et al. analyzed data from 7 observational studies (2673 patients—mostly elderly) and showed that preoperative vitamin D deficiency was associated with a 1,54-fold increased risk of POD or post-operative cognitive dysfunction. This study indirectly supports the preoperative supplementation of vitamin D in deficient patients prior to surgical procedures [67]. A systematic review by Iglar and Hogan investigated the impact of peri-operative vitamin D status on surgical outcomes. They analyzed 31 studies with a large proportion of patients who were 65, including various prospective, randomized, and retrospective approaches, examining the correlation between low vitamin D levels and post-surgical complications across different surgical procedures. The main finding was that 84% of the studies reported at least one significant adverse outcome associated with low vitamin D levels. The review supports the hypothesis that insufficient vitamin D levels are linked to poor post-operative outcomes, suggesting a potential benefit in optimizing vitamin D levels to enhance surgical recovery [68].

Finally, management of functional hypogonadism with testosterone therapy is a promising intervention that should be investigated in future studies [69].

### 6.5. Management of Cognitive Dysfunction and Mental Disorders

A systematic review by Zhao et al. focused on the effects of peri-operative cognitive function training on post-operative cognitive dysfunction and POD. The analysis involved 975 patients (the majority being 65+ years), with interventions including various forms of cognitive training, like memory exercises, problem-solving tasks, and other cognitive challenges, aimed at enhancing cognitive domains, such as memory, attention, and executive function. The cognitive function outcomes were assessed using tests like the MMSE and the MoCA. The results indicated that cognitive training led to a significant reduction in post-operative cognitive dysfunction by 50%. However, the incidence of POD did not show a statistically significant relationship [70]. 

A systematic review by Bowden et al. comprehensively examined cognitive interventions to improve cognitive function in adult surgical patients after general anesthesia. It incorporated 9 studies, predominantly randomized controlled trials (the majority of patients being 65+ years), which included interventions that were primarily aimed at enhancing memory and attention. The results showed a significant beneficial impact on memory, with a detailed analysis suggesting varying efficacy across other cognitive domains [71]. 

A study by Abraham et al. applied a peri-operative mental health intervention program aimed at older adults (with a mean age of 68 years) undergoing various surgeries, who also suffered from depression or anxiety. This intervention combined medication optimization with a wellness program adhering to behavioral activation principles. The program yielded promising results, demonstrating substantial engagement and completion (above 80%). Participants reported significant improvements in mental health, evidenced by decreased scores on the Patient Health Questionnaire for Anxiety and Depression. This study highlights that the instance of a major surgery could be a significant opportunity to optimize mental health in elderly patients [72]. 

### 6.6. Structured Rehabilitation Programs

Structured rehabilitation programs that combine nutritional support, exercise, physiotherapy, and cognitive interventions can potentially improve the post-operative outcomes for elderly patients. A study by Koh et al. evaluated the effects of a structured prehabilitation program for elderly patients undergoing elective colorectal surgery. This nonrandomized, prospective cohort study included 58 elderly patients (with a mean age of 78.5 years) who underwent a 3-week pre-surgery regimen including geriatric assessment, nutrition supplementation, and resistance training. Results showed a reduction in hospital stays by an average of 6.8 days, improved quality of life, and significant a savings in health costs. However, it did not significantly impact major post-operative complications or mortality [73]. 

The EASE-BE FIT pilot study aimed to develop a post-surgical reconditioning program for patients aged 65+ years who underwent emergent abdominal surgery. The program was implemented in the hospital setting and had a duration of three days, focusing on enhancing muscle strength, balance, and endurance. Participants in the BE FIT group performed better on the 30 s sit-to-stand test, improving by an average of 1.9 stands compared to usual care (*p* = 0.05). No adverse events were reported, indicating the safety and feasibility of the program [74]. 

The Enhanced Recovery After Surgery (ERAS) protocols were initiated in 2001 by a group of European surgeons and aimed to enhance the quality of surgical recovery. The ERAS approach is comprehensive, involving preoperative, intraoperative, and post-operative interventions to speed recovery and reduce complications. This includes strategies like preoperative nutritional support, optimal pain and fluid management, and early mobilization and exercise. Implementing these protocols has significantly reduced hospital stays by 30–50% and minimized complications, leading to substantial cost savings [75]. 

A systematic review and meta-analysis of randomized controlled trials demonstrated that a preoperative CGA can guide the selection of elderly patients for prehabilitation and post-operative interventions. The majority of studies included only patients aged 65+ years and applied interventions such as the Hospital Elder Life Program (HELP) or prehabilitation. Most studies in the analysis demonstrated positive outcomes, including reductions in post-operative complications, hospital LOS, reduced functional decline, and reduced rehospitalization rate [76].

A list of recommended peri-operative interventions in geriatric patients and their potential benefits is presented in Table 2.

## 7. Conclusions

In conclusion, the growing population of elderly patients undergoing surgical procedures presents unique challenges that cannot be adequately addressed by traditional preoperative risk assessment tools. The CGA emerges as a more holistic and reliable approach for evaluating the multifaceted needs of these patients. By incorporating key factors such as frailty, sarcopenia, nutritional status, cognitive function, and functional capacity, the CGA allows clinicians to predict post-operative complications more accurately and tailor interventions, including prehabilitation and targeted post-operative care, to improve outcomes. 

While implementing the CGA in everyday clinical practice might introduce complexities, its potential to improve peri-operative risk assessment and post-operative outcomes justifies its broader adoption. The two-step approach, involving initial screening followed by a full CGA in high-risk patients, offers a practical solution that balances clinical efficiency with comprehensive care. 

Future research should focus on further simplifying the CGA protocols for widespread use and developing evidence-based guidelines that can promote its implementation in diverse clinical settings. By continuing to improve the peri-operative geriatric care, we can maximize both quality of life and long-term outcomes of elderly patients.

## Figures and Tables

**Table 1:** Preoperative comprehensive geriatric assessment and its prognostics.

Components	Tools for Assessment	Prognostics
Frailty	▪ Fried Frailty Phenotype ▪ Clinical Frailty Scale (CFS) ▪ Essential Frailty Toolset (EFT) ▪ Rockwood Frailty Index	▪ Post-operative complications (up to 5-fold increased risk) ▪ Prolonged hospital length of stay (up to 3 days longer) ▪ Post-operative mortality (up to 8-fold higher 30-day mortality) ▪ Post-operative delirium (3-fold increased risk)
Sarcopenia	▪ Initial methods for assessment - Grip strength - Chair stand test ▪ Confirmation of diagnosis - Dual-energy X-ray absorptiometry - Bioelectrical impedance analysis - Imaging techniques (computed tomography, magnetic resonance imaging, ultrasound) ▪ Severity - Timed Up and Go Test (TUG) - Gait speed	▪ Post-operative complications (up to 4-fold increased risk) ▪ Prolonged hospital length of stay (up to 4.5 days longer) ▪ Post-operative mortality (up to 2-fold higher 30-day mortality) ▪ Increased needs for in-patient rehabilitation ▪ Increased post-operative inflammatory response.
Malnutrition	▪ Mini Nutritional Assessment (MNA) ▪ Malnutrition Universal Screening Tool (MUST)	▪ Post-operative complications (2.5-fold increased risk) ▪ Post-operative mortality (2-fold or higher rates) ▪ Post-operative independence (up to 1.5-fold increased risk)
Cognitive dysfunction	▪ Mini Mental State Examination (MMSE) ▪ Montreal Cognitive Assessment (MoCA) ▪ Clock drawing test	▪ Post-operative complications (1.5-fold increased risk) ▪ Post-operative delirium ▪ Post-operative mortality (1.5-fold higher 30-day mortality)
Functional status impairment	▪ Barthel Index ▪ Katz Index of Independence in Activities of Daily Living (ADL) ▪ Lawton Index of Independence in Instrumental Activities of Daily Living (IADL)	▪ Post-operative complications (3-fold increased risk) ▪ Post-operative delirium (2-fold increased risk) ▪ Prolonged hospital length of stay (up to 5 days longer)

**Table 2:** Recommended peri-operative interventions in geriatric patients.

Peri-Operative Interventions	Implementation	Benefits
Nutrition	▪ Adequate protein and caloric intake ▪ Supplementation with regiments high in protein and micronutrients in patients at risk for malnutrition	▪ Reduction in muscle atrophy ▪ Improvement in muscle strength ▪ Functional improvement ▪ Reduction in post-operative complications by up to 30% ▪ Improved wound healing
Exercise and physiotherapy	▪ Combination of aerobic and resistance training ▪ Physiotherapy focusing on the respiratory system, mobilization, balance, and functionality	▪ Improvements in physical capacity and muscle strength ▪ Improvements in quality of life ▪ Reduction in post-operative complications (up to 50% reduction in respiratory complications with physiotherapy) ▪ Reduction in hospital length of stay (up to 3 days shorter)
Correction of deficiencies	▪ Management of iron and vitamin D deficiency	▪ Iron supplementation: reduction in transfusion requirements ▪ Vitamin D supplementation: - Reduction in atrial fibrillation risk following cardiac surgery - Reduction in hospital length of stay - Possible reduction in post-operative delirium and post-operative cognitive dysfunction
Cognitive stimulation	▪ Memory exercises ▪ Problem-solving tasks ▪ Other cognitive challenges aimed at enhancing cognitive domains such as memory, attention, and executive function	▪ Reduction in post-operative cognitive dysfunction ▪ Possible reduction in post-operative delirium
Mental health optimization	▪ Psychotherapy combined with medications	▪ Improvement in quality of life ▪ Possible improvements in adherence with the physician’s post-operative instructions

## Data Availability

Data sharing is not applicable.
